# The effect of educational intervention based on health belief model on colorectal cancer screening behaviors

**DOI:** 10.1186/s12889-024-19180-8

**Published:** 2024-06-19

**Authors:** Tayebeh Rakhshani, Elham Razeghi, Seyyed Mansour Kashfi, Amirhossein Kamyab, Ali Khani Jeihooni

**Affiliations:** 1 Nutrition Research Center, Department of Public Health, School of Health, Shiraz University of Medical Sciences, Shiraz, Iran; 2 Department of Public Health, School of Health, Shiraz University of Medical Sciences, Shiraz, Iran; 3 Faculty of Medicine, Fasa University of Medical Sciences, Fasa, Iran

**Keywords:** Health Education, Health Belief Model, Behavior, Health-related behaviors, Screening, Colorectal Cancer

## Abstract

**Background:**

Colorectal cancer is the second most prevalent cause of death from malignancies globally. The present study was conducted targeting the influence of an educational intervention based on the health belief model (HBM) on colorectal cancer screening behaviors in people 50 years old and older.

**Methods:**

All 134 samples were included in this quasi-experimental study from Fasa City Health Service Center, equal halves were random into experimental group and control group. The data collection tool was a questionnaire that contained questions on demographic variables, knowledge, and HBM constructs (perceived sensitivity, perceived intensity, perceived benefits, perceived barriers, self-efficacy, and screening behaviors). Both groups answered the questionnaire before and two months following the intervention. There were six 90-minute instruction sessions for the intervention group. SPSS 22 and descriptive and analytical tests (independent t-test, paired t-test, and Chi-square test) were used for data analysis (*P* < 0.05).

**Results:**

59 women and 75 men took part in this study. A majority of participants were married and had at least high school diploma. The findings indicated that the mean scores for knowledge, each of the HBM’s constructs, and cancer screening behaviors did not differ significantly from one another before the intervention between the test group and the control group. However, post the intervention, the intervention group exhibited a significant rise in all mentioned dimensions.

**Conclusion:**

In light of the outcomes, the application of the HBM on colorectal cancer screening behaviors in people 50 years and older was successful. This approach might serve as a helpful foundation for planning, carrying out, and overseeing colorectal cancer screening programs.

## Background

Currently, one of the most significant health issues of the previous century is cancer and is responsible for one out of every eight deaths worldwide [1]. With approximately 1.8 million occurrences in 2018, colorectal cancer is among the most prevalent cancers worldwide [2]. The International Organization for Research on Cancer has calculated the prevalence of colorectal cancer in Iranian men to be 8.7 per 100,000 people with a death rate of 6.3 per 100,000 people and in women to be 6.4 per 100,000 people with a death rate of 4.6 per 100,000 people [3]. Every year, about one million people in the world are diagnosed with colorectal cancer, among which about half of them die within five years after the onset of the disease [4].

Fortunately, screening enables early detection of the disease in time before it progresses further. Colorectal cancer screening programs increase the 5-year survival rate to about 90%. As a result, colorectal cancer screening is advised and acknowledged by the World Health Organization and health authorities as a successful strategy to lower incidence and death [2]. Due to recent increases in colorectal cancer incidence, and considering the fact that a great portion of colorectal cancer cases occur in people older than 50, it is necessary for people over 45 years old to undergo an annual fecal occult blood test and periodical proctosigmoidoscopy every 3 to 5 years [5]. These practices help the diagnosis of precancerous polyps, so that they can be removed before they turn into cancer [6]. Previous evidence have showed that knowledge, attitude, and beliefs regarding risk factors and diseases is highly related to screening behaviors, and education has found to be effective in creating healthy behaviors, a better understanding of the disease, and preventing or delaying complications [7].

Considering the complexity of screening behaviors, the use of theories and behavioral patterns in conducting screening tests has become necessary over time [8]. Effectiveness of the health education programs depends to a large extent on the use of proper theories and models [9]. The Health Belief Model (HBM) is one of the oldest health behavior theories that has been used for almost half a century with great success in various subjects. This model shows how beliefs and behavior are related, and is commonly used to assess health beliefs regarding screening behaviors [10]. It assumes that behavior is a function of knowledge and attitude, and correct perceptions will change a behavior. Hence, this model plays a great role in disease prevention. The structures of this model include perceived sensitivity, perceived severity, perceived benefits, perceived barriers, cues to action, and self-efficacy [11].

In accordance with this model, in order to perform colorectal cancer screening, individuals must first perceive the risk of developing its complications (perceived sensitivity), then comprehend the extent of this risk and its seriousness in their physical, psychological, social, and economic dimensions (perceived severity), with the help of encouraging cues from their internal environment or the environment around them (cues to action); perceive the action as less expensive than its benefits (perceived barriers); anticipate positive outcomes and advantages that they believe they will gain by participating in regular screening tests (perceived benefits); and have faith in their own abilities to carry out the behavior (self-efficacy) in order to ultimately engage in preventive behavior [12]. This model can be useful in understanding the facilitators and barriers for colorectal cancer screening [13]. Considering the alarming incidence rates of colorectal cancer, the important role of timely prevention in reducing the complications and its mortality, and the effectiveness of the HBM in the preventing cancers, this research was aimed to determine the effects of an educational intervention based on the HBM on colorectal cancer screening behaviors in a group of people over 50 years of age.

## Methods

### Research design

The intended quasi-experimental study investigates 134 people who were referred to Urban Health Service Center of Fasa City in 2022. The sample size was obtained using the mentioned formula. To determine the sample size, the formula for comparing two dependent means was used in the Stata software version 11 environment. According to Gholampour et al.‘s study, the required sample size in each group was 67 people [14].


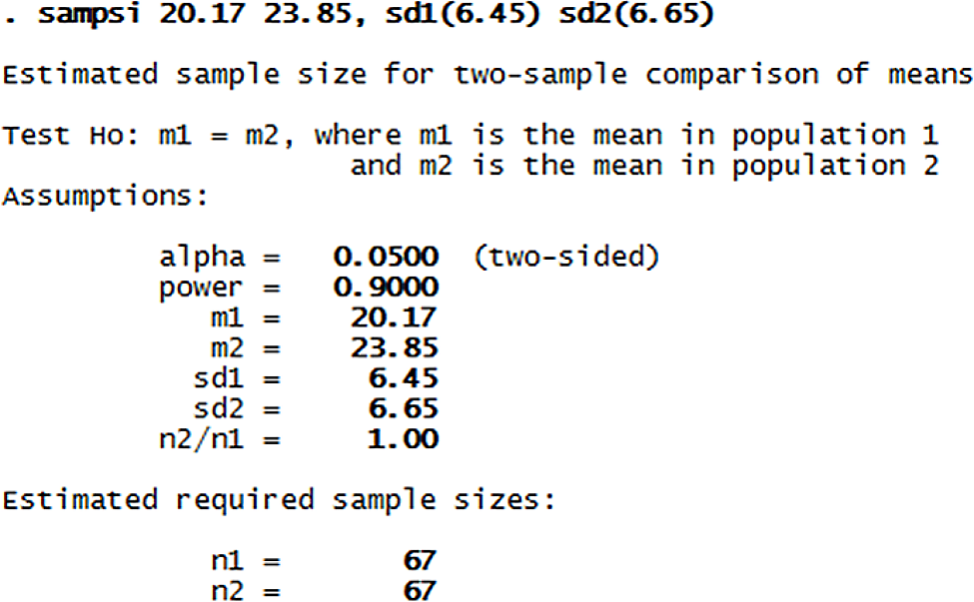


According to the sample size formula, 134 people who referred to Urban Health Service Center of Fasa City in 2022 who met the inclusion criteria were selected randomly. Then, using a random number table (even numbers for the control group, and odd numbers for the experimental group), the participants were split into these two groups (67 people in each group).

### Inclusion and exclusion criteria

The inclusion criteria were age over 50 years, having no history of previous screening tests, no history of colorectal or colon cancer, interest in participation in the research, and continuous and consecutive attendance in the educational program. The exclusion criteria were more than one session absence from the classes, not filling any of the questionnaires, and changing the place of residence during the study.

### Protocol

After specifying the study groups in 12 February 2022, an initial evaluation of the participants was performed using the questionnaires, and their scores were recorded. Then, the experimental group was split into six groups of ten to eleven individuals. The classes were held for each of the six groups in one day a week, and phone calls were used to announce the day and hour of the meetings, from 28 February till 7 April 2022. Educational booklets, posters, PowerPoint presentations, and videos were used as educational aids. One of the sessions was devoted to group discussion because it is an effective teaching strategy for small groups and is frequently employed in the domains of problem solving and attitude modification.

According to the results of the pre-test phase, it was found that the knowledge scores indicated poor knowledge, and it can be said that it was necessary to give accurate information to the people, which justifies the necessity of training directly and face-to-face in health centers. The program lasted for six weeks for the experimental group. Two months after the end of the training in 9 June 2022, a second exam was administered to both research groups using the identical original questionnaire. During the entire intervention, the control group was unaware of the implementation of the related training program. However, in order to follow ethical considerations, the same intervention materials was provided for the controls after the study’s completion. The content and training methods of the sessions are as described in the following table (Table 1).

**Table 1:** Educational contents discussed during the educational program

Meeting	Instructional materials	Teaching strategy
First meeting	To enhance the participant’s understanding of colorectal cancer, the subsequent points were observed: - Outlining the generals, the study’s goals, and the screening plan’s advantages - The necessity and significance of understanding colorectal cancer symptoms, prevention strategies, screening procedures, and occult blood tests in stool	Question and answers Group discussions Face-to-face training
Second meeting	The following subjects were brought up in order to elevate the constructions of perceived sensitivity and intensity: - The significance of a latent gene (perceived susceptibility) and the lack of a positive family history of colorectal cancer -The disease’s potential hazards, complications, and seriousness, death rates from late-stage colorectal cancer diagnosis -Complications and repercussions of late-stage diagnosis (perceived severity) in the absence of clinical symptoms	Question and answers Group discussions
Third meeting	To enhance the perceived benefits and perceived barriers, the subsequent subjects were brought up: - The financial, psychological, and physical toll that colorectal cancer takes, as well as the advantages of preventing it - The embarrassment and shame that screening procedures cause - The value and advantages of screening	Question and answers Group discussions
Fourth meeting	In order to focus on self-efficacy, the following observations were made: -Fear about people’s potential dangers and uncertainties, such as occult blood tests in stool - Restrictions on food and medication prior to the test -When and how to collect stool samples and send them to the lab	Question and answers Group discussions
Fifth meeting	The following issues were brought up to affect the cues to action’s structure: - Support, assistance, and direction with screening - Motivating and convincing them by reminding them of things	Question and answers Group discussions Sending text messages
Sixth meeting	An overview of earlier meetings	Question and answers Group discussions Face-to-face training

### Data collection tool

The tool for data collection was created through a collaborative process involving input from subject matter experts and pilot testing among the target population to ensure validity and reliability. The questionnaire content included four main domains: (a) Socio-demographic variables (b) Knowledge (c) Health Belief Model (HBM) (d) Behavior.

### Socio-demographic variables questionnaire

The questionnaire of demographic variables included age, sex, marital status, level of education, occupation, and socioeconomic status.

### Knowledge questionnaire. - add the title “Health Belief Model (HBM) questionnaire

#### Knowledge

This section contained 13 five-choice questions related to colorectal cancer screening behaviors, with answers from strongly agree to strongly disagree, with minimum and maximum scores of 13 and 65. Obtaining a higher score indicated more knowledge in this regard.

#### Perceived sensitivity

This part included seven questions with a 5-point Likert scale (from strongly agree to strongly disagree) regarding colorectal cancer screening behaviors in men and women over 50 years old. The minimum and maximum scores obtained were 7 and 35, where scores between 0 and 14 indicated low level, 15 to 24 were medium level, and 25 to 35 showed high level of perceived sensitivity, and higher scores indicated high perceived sensitivity of colorectal cancer screening behaviors.

#### Perceived intensity

This section included six questions with a 5-point Likert scale (strongly agree to strongly disagree) regarding the perception of colorectal cancer screening behaviors. The minimum and maximum scores obtained for this section were 6 and 30. 0 to 10 showed a low level, 11 to 20 showed a medium level, and 21 to 30 showed a high level of perceived intensity.

#### Perceived benefits

It included 5 questions with a 5-item Likert scale (strongly agree to strongly disagree) regarding the perception of benefits obtained from colorectal cancer screening behaviors. The minimum and maximum scores for perceived benefits were 5 and 25, with scores between 0 and 10 being low, 11 and 20 being medium, and 21 and 25 being high levels of perceived benefits.

#### Perceived barriers

It included 12 questions with a 5-point Likert scale (strongly agree to strongly disagree) regarding the perception of colorectal cancer screening behaviors. The minimum and maximum scores obtained for perceived barriers were 12 and 60. 0 to 20 showed low levels, 21 to 40 medium levels, and 41 to 60 high levels of perceived barriers.

#### Cues to action

Two multiple-choice questions were used to examine this structure: which resources are available for following health guidelines and conducting screening tests (external cues to action) and which symptoms prompted you to do a fecal occult blood test (internal cues to action). These scores were between 0 and 6.

#### Perceived self-efficacy

This section included five questions with a 4-point Likert scale (agree, have no opinion, disagree, strongly disagree) regarding people’s ability to express their behavior regarding colorectal cancer screening. The minimum and maximum scores obtained for self-efficacy were 5 and 20. 0 to 7 showed a low level, 8 to 14 showed a medium level, and 15 to 20 showed a high level of perceived self-efficacy.

#### Behaviors questionnaire

This part was assessed through 11 questions on a 5-point Likert scale (strongly agree to strongly disagree) regarding colorectal cancer screening behaviors. The minimum and maximum scores obtained for behavior are 11 and 55. 0 to 20 showed a low level, 21 to 40 showed a medium level, and 41 to 55 showed a high level of this variable.

### Validity of the questionnaire

The validity of the questionnaire was done in both quantitative and qualitative ways. Ten professors with expertise were given the questionnaire for the qualitative section. Content validity index (CVI) and content validity ratio (CVR) were employed to examine the quantitative portion. The board of experts measured the CVI in order to calculate it. Each item was ultimately found to be greater than 0.62 using the Lawshe table index, and the questions pertaining to that item were deemed necessary and retained for more examination [15].

In this study, we used CVI to provide a quantitative measure of agreement among experts regarding the necessity of each item. It ensures that the items are appropriate for measuring the construct of interest. CVI helps ensure that the content of the questionnaire adequately covers all aspects of the construct being measured, enhancing its content validity.

On the other hand, CVR evaluates the necessity of each item in a questionnaire by asking experts to judge whether an item is essential for measuring the construct. It assesses the essentiality of items rather than their relevance or clarity. CVR helps to identify items that may not be crucial for measuring the construct and may need to be revised or removed to improve the questionnaire’s quality and improving the efficiency of data collection.

Relying solely on CVI may overlook items that, while relevant and clear, might not be essential for measuring the construct. These items could potentially inflate the length of the questionnaire without contributing significantly to its validity. On the other hand, relying solely on CVR may miss items that are relevant and clear but are mistakenly judged as non-essential by expert raters. These items may still be important for capturing nuances or comprehensiveness within the construct being measured.

Internal consistency techniques were applied to gauge the tool’s reliability. The questionnaire was given to 30 participants who met the study’s eligibility requirements in order to ascertain the internal correlation of the tool’s various components. Following analysis using SPSS 24, the alpha coefficient Cronbach’s was established for each factor. The reliability of perceived sensitivity was confirmed to be 0.72, perceived intensity 0.71, perceived benefits 0.89, perceived barriers 0.73, self-efficacy 0.77, cues to action 0.73, and behavior 0.78. Considering that the Cronbach’s alpha values calculated for each of the dimensions and constructs studied in this research were greater than 0.7, the reliability of the tool was evaluated and confirmed as acceptable.

### Statistical analysis

In this research, mean and standard deviation used to measure continuous variables, while Chi-square is employed to measure categorical variables. Additionally, independent T-test and paired t-tests were used to assess differences, and a significance level of *p* < 0.05 was adopted. Data was analyzed using SPSS 22.

## Results

59 women and 75 men took part in this study. A majority of participants were married and had at least high school diploma. Table 2 compares the intervention and control groups in terms of their demographic variables. The results show that the two groups were not significantly different from each other in terms of age (*P* = 0.508), sex (*P* = 0.508), marital status (*P* = 0.566), level of education (*P* = 0.057), occupation (*P* = 0.150), and economic status (*P* = 0.120).

**Table 2:** Demographic variables of the participants

Variable		Experimental group (*n* = 67)		Control group (*n* = 67)		*P*-value
		Number	Percentage	Number	Percentage	
Age		56.20 ± 5.23		55.68 ± 3.72		0.508*
Sex	Female	27	40	32	48	0.508**
	Male	40	60	35	52	
Marital status	Single	0	0	0	0	0.566**
	Married	60	90	61	91	
	Widowed	7	10	6	9	
Level of education	Lower than high school diploma	17	25	13	19	0.057**
	High school diploma	18	27	20	30	
	University	32	48	34	51	
Occupation	Self-employed	50	75	45	67	0.150**
	Employee	17	25	22	33	
Monthly household income	< 20 million Rials (< 32$)	1	1	1	1	0.120**
	20–40 million Rials (32–64$)	26	39	30	45	
	> 40 million Rials (> 64$)	40	60	36	54	

* Independent t-test

** Chi square test

According to the results, before the intervention, there was no significant difference between the mean scores of knowledge, the HBM constructs, and colorectal cancer screening behaviors between the two study groups, but after the intervention, there was a significant difference among the two groups regarding the HBM constructs (*P* < 0.05). Moreover, contrary to the controls, a notable difference was found in each of the mentioned variables the experimental group post intervention (*P* < 0.05) (Table 3).

**Table 3:** Comparison of mean scores of knowledge, constructs of the HBM, and behavior in two study groups before and after the educational intervention

Variable	Group	Before intervention (Mean ± SD)	After intervention (Mean ± SD)	*P*-value*
Knowledge	Experimental (*n* = 67)	15.59 ± 1.62	61.05 ± 1.25	< 0.001
	Control (*n* = 67)	15.91 ± 1.16	16.08 ± 1.12	0.150
	*P*-value**	0.432	< 0.001	
Perceived sensitivity	Experimental (*n* = 67)	14.24 ± 2.94	31.25 ± 1.12	< 0.001
	Control (*n* = 67)	15.57 ± 2.31	15.53 ± 2.35	0.321
	*P*-value**	0.063	< 0.001	
Perceived severity	Experimental (*n* = 67)	10.35 ± 2.78	26.24 ± 1.82	< 0.001
	Control (*n* = 67)	10.31 ± 2.66	11.35 ± 2.67	0.321
	*P*-value**	0.928	< 0.001	
Perceived benefits	Experimental (*n* = 67)	9.85 ± 2.27	22.50 ± 1.38	< 0.001
	Control (*n* = 67)	9.32 ± 1.82	10.38 ± 1.87	0.208
	*P*-value**	0.121	< 0.001	
Perceived barriers	Experimental (*n* = 67)	52.09 ± 2.04	19.97 ± 1.66	< 0.001
	Control (*n* = 67)	52.91 ± 2.06	53.37 ± 2.37	0.062
	*P*-value**	0.087	< 0.001	
Perceived self-efficacy	Experimental (*n* = 67)	8.59 ± 1.80	17.99 ± 1.08	< 0.001
	Control (*n* = 67)	8.70 ± 1.79	8.80 ± 1.55	0.163
	*P*-value**	0.714	< 0.001	
Cues to action	Experimental (*n* = 67)	2.01 ± 0.02	4.12 ± 0.30	< 0.001
	Control (*n* = 67)	2.31 ± 0.11	2.83 ± 0.16	1.000
	*P*-value**	0.310	< 0.001	
Behavior	Experimental (*n* = 67)	15.12 ± 1.43	50.42 ± 1.08	< 0.001
	Control (*n* = 67)	15.05 ± 2.20	16.65 ± 1.31	1.000
	*P*-value**	0.413	< 0.001	

* Paired t-test

** Independent t-test

## Discussion

The purpose of this study was to find out how an education based on the HBM could affect the screening behaviors of colorectal cancer in in people 50 years old and older who were referred to Fasa, Iran health centers in 2022. The mean scores for preventative behaviors were not at a high level, according to the data. Given the alarming rate of colorectal cancer incidence, and the role of screening in its survival, making sure that people seek medical attention for questionable symptoms and take advantage of screening programs are two important strategies to lower the death rate from cancer [12]. The absence of educational resources and the facilities required to take part in colorectal cancer screening programs may be among the main causes of people’s ignorance of colorectal cancer screening and their lack of involvement [16].

According to the findings, a notable distinction was observed between the mean scores of knowledge before and after the intervention within the experimental group, which shows the effectiveness of education in raising the knowledge of the participants. The role of radio and television, healthcare staff, and doctors in informing and guiding people to perform screening tests and training sessions for the prevention of colorectal cancer is very essential [17]. Hence, explaining the symptoms and ways of prevention by screening and performing an occult blood test through question and answer, group discussion, and face-to-face communication can be very effective. In addition, conducting interventions at the community level and retraining programs for healthcare personnel can also be very effective in reducing the burden of this disease [17]. In Lin et al.‘s study, people’s lack of knowledge about colorectal cancer decreased the screening rates markedly [18]. Therefore, appropriate knowledge in the studied population is a determining factor in the desirability of preventive behaviors.

Our results showed that the perceived sensitivity mean scores in the experimental group almost doubled after the intervention, which can be due to the novel techniques of teaching and the complete understanding of the subject by the audience. Other studies also showed similar results in line with the present study. In fact, based on the HBM, it can be concluded that if a person believes that they are exposed to a chronic disease such as cancer (perceived susceptibility), the seriousness and the complications of the disease is more understandable for them (perceived severity) [17]. The comparison of the findings indicates the importance of conducting effective educational interventions in increasing people’s sensitivity to the risk of cancer, and the more sensitive a person is to the risk of contracting the disease, the more conscious they will be of the risks of confronting that disease.

The perceived severity mean scores of the participants in the experimental group have increased sharply after the intervention. This shows that the training of colorectal cancer screening behaviors based on the HBM has led to an improvement in increasing the perceived severity of the participants. According to Becker, a person’s perception of the perceived severity of the disease’s consequences affects their intention for self-care [19]. In fact, this model suggests that increased self-awareness about one’s health risks encourages individuals to engage in preventive behaviors [13]. In line with our results, the findings of the former studies confirm the efficacy of this method in increasing the perceived severity structure [20].

Taking a look at the results, the experimental group manifested ample hike in their mean perceived benefits scores. Probably, the instruction of screening behaviors, the expression of the physical, psychological, and financial losses associated with colorectal cancer and the benefits of its prevention, the advantages of early diagnosis and more effective treatments, and raising their knowledge of polyps as benign cells that grow slowly but have the potential to become malignant and the significance of screening tests to find and remove them before they become malignant all contributed to their perceived benefits. Consistent with previous research findings, the majority of participants in the current study did not show acceptable levels for perceived benefits, as indicated by their mean score [21]. The mean perceived benefit ratings in these trials showed that the subjects did not have a strong knowledge of the advantages of taking preventative action against colorectal cancer, nor did they have a strong understanding of the importance of routine check-ups. It is evident from other studies that the perceived benefits scores were not accepted, indicating that limited knowledge and information about this illness was unable to alter the examined populations’ attitudes and views about the advantages of engaging in preventative practices [14].

Our study’s findings indicate that there were major barriers preventing people from engaging in colorectal cancer screening preventive activities before the intervention, which reduced largely after the intervention in the experimental group. The larger the perceived restrictions, the lesser an individual’s comprehension of the hazards associated with acquiring an illness [22]. The fact that the study participants felt they had less difficulty adopting preventative practices post-intervention makes the low level of perceived barriers a plus. Thus, as this study showed, it is possible to minimize the barriers to engaging in behaviors as much as possible by implementing a number of interventions and forecasting suitable regulations. Other studies also reported same findings in their participants [13, 21].

One of the other variables that has almost doubled in our study in the experimental group was the self-efficacy mean scores. Self-efficacy is an important prerequisite for self-management to change behavior, which can promote healthy behaviors. In this study, the role model used to improve the self-efficacy for the experimental group has been used in other similar studies [23]. Qian et al. obtained a similar result in this regard and stated that in patients with gastrointestinal cancers, increased self-efficacy is associated with lower depression, higher social support, and higher vitality, all of which can lead to greater gastrointestinal cancer control [24].

Our results showed that the cues to action in the experimental group almost doubled after the intervention. In fact, the reason for this increase can be attributed to the encouragement, help, and guidance for screening, encouraging and motivating people by sending reminder messages, and having group discussion and question and answer sessions. On social networks, it is possible to answer all the questions raised on a scientific manner, distribute educational booklets and pamphlets containing the main contents of the sessions, and arrange face-to-face counseling sessions. Various studies have shown that recommendations from doctors, family, and friends were the most common reasons for colorectal cancer screening [25]. These findings emphasize the important role that doctors play in cueing action [26]. Although studies reposted that the guidelines used for each country are better defined locally, we can use the experience of the actions of other countries to implement them regionally [27].

Also, the training provided had substantially improved the screening of colorectal cancer behavior of the experimental group. According to health experts, a person’s understanding of the benefits of behavior can facilitate behavior change [28]. In fact, the more people realize that changing their behavior is more beneficial for themselves, the more willing they will be to change their behavior, which can be considered beneficial for people with gastrointestinal cancers [29]. In this regard, it is necessary to mention that self-efficacy is considered an important precondition for behavior change, and as mentioned above, in the present study, the level of self-efficacy in the experimental group improved remarkably, almost two times after the intervention.

### Limitations

One of the limitations of this study was the behavior of the participants in the research, which was inevitably collected through self-reporting. Since the sampling was performed only in one city, it may not be generalizable to the whole community. Therefore, it is suggested to carry out other research with a follow-up period to determine the extent of the long-lasting effects of education based on the HBM on colorectal cancer screening behaviors, and also to conduct similar research in other cities and regions to provide a suitable treatment field for the general public.

## Conclusion

The study’s overall findings demonstrate a significant difference in the intervention group’s mean knowledge scores as well as scores on all of the HBM’s constructs (perceived sensitivity, perceived intensity, perceived benefits, cues to action, self-efficacy, and behavior) following the completion of the educational program. This suggests that the training program that was put into place had a positive effect. As a result, it can be said that, in contrast to the control group, the research participants benefited greatly from training based on the HBM. Therefore, methods of increasing these structures, such as verbal persuasion, increasing awareness of people’s benefits, obstacles, and abilities through virtual media, and providing suitable models for them, can be used as factors promoting colorectal cancer screening behaviors.

Taking a look at the results, by promoting early detection and prevention through targeted educational programs, healthcare providers not only can improve public health outcomes and quality of life for older adults, but the can also reduce the burden on the healthcare system and healthcare providers by preventing the disease, instead of the well-known surgical, chemical, or immunological therapies. Additionally, this study highlights the importance of utilizing behavior change theories, such as the HBM, to design effective interventions that address individual beliefs and attitudes towards cancer screening.

## Data Availability

The datasets used and/or analyzed during the current study are publicly available from the corresponding author on reasonable request.
